# Bilateral effect of aging population on consumption structure: Evidence from China

**DOI:** 10.3389/fpubh.2022.941485

**Published:** 2022-08-25

**Authors:** Yue Wang, Wan Zhao, Wanrong Meng

**Affiliations:** School of Public Management, Liaoning University, Shenyang, China

**Keywords:** aging population, upgrade of the consumption structure, bilateral stochastic cutting-edge model, panel data, regulate economy

## Abstract

The deepening of aging population inevitably in China will exert a far-reaching influence on national consumption and economic transformation. Based on interprovincial panel data in 2000–2018, this paper measured the ratio of five survival and enjoyment consumptions in disposable incomes, reconstructed the indicators for upgrading the consumption structure, used the bilateral stochastic cutting-edge model, and decomposed the aging population to realize the net effect from the positive and negative effects generated by the consumption structure. The findings indicated that (1) aging population played a positive and negative bilateral effect on upgrading the consumption structure, in which the positive effect upgraded 14.04% of the consumption structure while the negative effect degraded 6.18% of the consumption structure. The comprehensive net effect upgraded 7.86% of the consumption structure. (2) From the perspective of the time effect, under the positive and negative effects of aging population, the consumption structure was upgraded 7.861% on average every year. (3) Regarding the regional effect, the promotion role of aging population was the highest in the eastern region, followed by the west. The middle was the lowest. By combining with estimation results of each province, the promotion role brought by aging population in the northeast and southwest was lower. Based on the above-mentioned research results, this paper proposed some advice for positively developing silver hair economy, promoting the improvement on the consumption structure according to circumstances, developing the perfect aging consumption market, exploring the consumption potential of the elderly, accelerating the urbanization development progress, and stimulating consumption growth relying on the Internet.

## Introduction

To dialectically consider the effect of aging population, exploring how to relieve the demographic structure dividend under the background of keeping deepening aging population, promoting growth of domestic demands, and accelerating the domestic circulation, the following questions should be answered: first, what is the effect of aging population for the upgrade of the consumption structure; second, what is the size of aging population on the upgrade effect of the consumption structure; third, what are time distribution characteristics and regional distribution features for the upgrade effect of aging population consumption structure. On this basis, this paper selected demographic and consumption data of 31 provinces in 2000–2018 and explored the upgrade effect of the aging population consumption structure, for the sake of proposing some effective advice.

In 2020, the “14th 5-Year Plan” specially proposed to depend on the powerful domestic market, insist on expanding domestic demands, and accelerate the domestic circulation. Meanwhile, China's population structure is undergoing a rapid transformation. In 2021, the 7th National Population Census data showed that the population of China over 60 years old occupied 18.70%, in which the population over 65 years old accounted for 13.50%. Compared with 2010, the ratio of older adults over 60 years old increased 5.44% during 10 years. At the same time, this ratio keeps growing. Relevant experts predict that in 2040, the elderly population over 65 years old in China will reach 27.8% and this value will be up to 32.2% in 2060 (1). Under the context of keeping increasing the elderly, it is of great significance to economic development by identifying whether it can effectively promote consumption growth and accelerate consumption transformation. The CPC Central Committee's Proposal on Formulating the 14th 5-Year Plan for National Economic and Social Development and the Long-term Goals for 2035 passed in the Fifth Plenary Session of the 19th Central Committee also emphasizes on positively implementing the national strategy to cope with aging population, positively changing aging risks into the “longevity dividend,” accelerating the new industry, new state, and new pattern of silver hair economy, expanding silver hair consumption, continuously expanding domestic demands to enrich the domestic circulation, promoting the benign interaction of international double circulation, and cultivating the new power of economic growth.^1^

However, the current Chinese residents' consumption rate was present in the long-term downtrend. During the period 1978–2010, residents' consumption rate was reduced by percent points (2). At the same time, in 2019, the part with the biggest occupation in China's consumption structure still included food expenditure, occupying 28.22%, followed by housing expenditure which occupied 23.45%. The ratio of such expenditures has already exceeded 50% of the total expenditure. The total expenditure proportion of five terms including traffic communication, household equipment and energy service, medical healthcare, educational leisure, and entertainment, as well as services accounted for 42.13%. The consumption expenditure ratio of the five terms excluding basic necessities in the UK, Denmark, Finland, Japan, and the USA in the same period has already exceeded 50%. As a whole, the overall consumption structure of China should be improved. The resident consumption rate should be improved. Meanwhile, with the deepening of aging, on the one hand, aging population keeps growing, which undoubtedly will accelerate demands for medical healthcare, services for the aged, and facilities.

This is an important opportunity to promote the upgrade of China's consumption structure. On the other hand, aging population means the population reduction of laborers. The reduction of primary groups is not good for consumption growth and consumption transformation. As a result, the influence of accurately estimating aging population on the transformation of the consumption structure will better serve economic development.

## Literature review

As an important aspect of economic growth, consumption is always the research key for domestic scholars. Particularly, with the growth of China's economy, research perspectives of scholars keep enriching from the consumption level and consumption structure to the upgrade of the consumption structure. According to existing studies, the main studies for the upgrade of the consumption structure are mainly concentrated on two aspects: On the one hand, they focus on discussing the current upgrade state of the consumption structure in China. Han and Xia (3), Shi et al. (4), and Gu and Xia (5), respectively, measured the upgrade state of the consumption structure in China from the perspectives of “developmental coefficient,” consumption structure, and consumption habits, as well as the framework of “survival type—developmental type—enjoyment type,” and demonstrated that the current consumption structure of China remained the upgrade state. Ye and Tang (6) further used the entropy weight method to measure the consumption upgrade index of each province in China and found that there was an obvious difference between regions. The provincial consumption upgrade index was successively decreasing from the east to the west. On the other hand, they concentrate on exploring the factors affecting the upgrade of the consumption structure. At present, scholars mainly study their relationship from the perspectives of social capital and consumption behavior (7), financial constraint (8), and Internet (9). However, so far, there have been fewer references analyzing the factors affecting the upgrade of the consumption structure from the demographic perspective. Particularly, with the constant deepening of aging population, the population structure transformation undoubtedly will exert an important influence on the upgrade of the consumption structure, showing the important significance to study their relationship.

First, aging population implies an increase in the elderly population ratio. The group is equipped with the significant features in consumption. As a whole, the consumption demands of the elderly in China are present in the trend of specialization and diversification. Moreover, the external dependency is gradually enhancing (10, 11). Regarding consumption demands, older adults tend toward medical treatment and caring services. Moreover, the consumption concept of older adults is relatively mature and rational (12). The overall consumption level of the elderly is lower, which is even lower than the national average level (13). What is more, there is an obvious difference in the consumption quantity between urban–rural older adults (14); in terms of the consumption structure, after becoming elderly, the food expenditure ratio of older adults is rising (13). The expenditure of clothing expenditure and traffic consumption is present in the declining trend (15). The expenditure of medical healthcare is significantly growing (16). The special consumption features of the elderly undoubtedly will generate the special influence on the upgrade of the consumption structure.

From the combination between aging and consumption, the scholars Grunberg and Modigliani (1954) (17) put forward the life cycle theory and discussed the relationship between the population structure variation and consumption. This theory argues that rational consumers will be based on the utility maximization principle to deal with savings and incomes at different age stages. With the constant enhancement of China's aging and the consumption level, Chinese scholars also conduct lots of studies from the perspectives of aging and consumption structure. First, in terms of research methods, Li and Gao (18), Cha and Zhou (19), Yu and Sun (20), and Bao and Li (21) made use of the gray system theory and method to verify whether aging population exerted an influence on the consumption structure, but they did not specially clarify the role direction of aging population. After that, scholars established the unitary linear model based on the life cycle theory to demonstrate the effect direction of aging population. Speaking of the research framework, the current studies are based on the following three frameworks. The first one is based on eight consumption types used by the National Bureau of Statistics to measure the consumption upgrade effect of aging population. For example, based on panel data of 30 provinces in China, Zhang et al. (22) redivided eight consumptions^2^ into “food and clothing consumption” and “other consumptions” to study the consumption upgrade effect of aging population. The second one is based on the survival materials, developmental materials, and enjoyment materials divided by Engels to measure the upgrade effect of the consumption structure for aging population. For instance, (23) measured the ratio of developmental and enjoyment expenditure in the total expenditure. Kou and Zhang (24) were based on the consumption framework of the type including survival—developmental—enjoyment. Tian (25) divided consumption into the health type, enjoyment type, and developmental type to measure the consumption upgrade effect of aging population. The third one is based on Stone's LES model and divided consumption into the basic consumption and developmental consumption to measure the upgrade effect of the consumption structure for aging population. For instance, Li (26) utilized the extended linear expenditure system (ELES) to divide the expenditure term in resident consumption structure into general commodities, and current research conclusions are mainly concentrated on the following two aspects: First, aging population is good for consumption structure upgrade (22–24); second, aging population is not good for upgrading the consumption structure (21, 25). Fewer scholars showed that the influence of aging population on the upgrade of the consumption structure was less significant (26).

On the whole, main conclusions of existing references for the upgrade effect of the consumption structure for aging population are mainly concentrated on the positive or negative unilateral effect but they have not noticed the simultaneous positive and negative bilateral effect for aging population changing with time and economic development. In the meanwhile, there are no relevant studies on time heterogeneity presented by aging population changing with time. In studies of different areas, scholars often make a comparison according to the eastern, middle, and western areas but cannot display the specific aging population effect of each province. Under the circumstance, through the bilateral stochastic boundary model, this paper conducted the estimation comparison on the positive and negative effects of aging population for the upgrade of the consumption structure and conducted quantitative estimation for the possible positive and negative effects on this basis, so as to evaluate the comprehensive influence of aging population on the upgrade of the consumption structure. What is more, this paper estimated the time development trend of the net effect for aging population and the upgrade effect of the independent consumption structure in each province, so as to propose more practical countermeasures.

## Theoretical assumptions

After entering 21st century, China starts entering the aging society, and the aging degree keeps deepening. This aspect is attributed to the reduction of the young population and the increase in the elderly population brought by the reduction of birth rate and death rate, showing the aging population. Beyond that, due to expected lifetime dilation of population, the existing population age keeps growing. The number of the elderly in society is present in the gradually growing trend. The role of factors in two factors ultimately will increase the elderly population ratio. The social aging degree keeps deepening, while the change in both aspects will exert multiple influences on consumption.

On the one hand, aging population is possible to bring the promotion effect for the upgrade of the consumption structure: (1) The life cycle theory argues that most people prefer the lifetime balance consumption. The redundant income in the young stage will be often used for paying debts in their youth or saving money for pension. Hence, when the ratio of elderly population in society is increasing, the consumption of entire society tends to increase. (2) At the same time, after labor population quits the labor force market, they have more time to enjoy leisure time so that the opportunity cost of enjoying leisure will be reduced, so as to promote the increase in enjoyment consumption for such a group, driving consumption upgrade. (3) With the increase in age, people's body function keeps degeneration while people's self-caring ability keeps weakening. This inevitably will drive the demands for medical treatment, pension, and elderly caring. The deepening of aging undoubtedly will drive the social consumption in medical healthcare and aging service consumption. (4) After post-60s and post-70s generations gradually become elderly, the consumption concept of older adults will tend to premature consumption and enjoyable consumption. (5) The extensive use of the Internet will make consumption more convenient, reducing consumption barriers caused by inconvenient trip. Besides, the upgrade of the consumption structure brought by aging population may display the obstruction effect: (1) Aging population means that the ratio of young people in the total population is reduced, while young people are social labor participants and main consumer groups. The ratio reduction of young people inevitably will reduce each consumption part. (2) Expected lifetime keeps lengthening, so older adults should allocate the longer time period to realize utility maximization and reduce the current consumption expenditure. Meanwhile, the willingness of preventive savings in their youth will be increased, while they will reduce the current consumption for elderly pension. (3) With the growth of age, after they leave the operating post, people will have more time to make a comparison on the purchased commodities. Older adults tend toward purchasing commodities with high-performance cost ratio while reducing the impulsion consumption. (4) With age, people's ability to accept and learn new things is weakening while the consumption mode keeps updating and becomes more intelligent. The lagged consumption mode affects the diversified development of the consumption structure. (5) The older generation is affected by their long-term life habits, so their overall saving conception is stronger and their consumption willingness is lower. On this basis, this paper proposed the hypothesis 1: Aging population showed the positive and negative bilateral effect on the upgrade of the consumption structure.

Meanwhile, since the reform and opening-up, China has undergone earth-shaking changes in economy, politics, and culture. People's disposable incomes keep increasing. Relative to 2013, per capita disposal incomes of China in 2020 were increased by 75.79%; the industrial structure kept optimization. In 2000–2019, the GDP ratio of the primary and secondary industries was, respectively, reduced by 7.11 and 38.97% from 14.67 and 45.54%. The GDP ratio of the tertiary industry was increased to 53.92% from 39.79%; people's consumption concept kept changing, while the consumption environment was constantly optimizing. On this basis, this paper put forward the hypothesis 2: The positive promotion effect of aging population for the upgrade of the consumption structure will perform the trend of increasing over time.

China has a vast territory, and each area has significant differences in economic development and population structure. In this way, the upgrade effect of the consumption structure brought by aging population in each region inevitably shows some differences. As a whole, the overall aging population degree in east China is higher, while the economic development level is also higher. The consumption market development is relatively sound, and high-tech application degree is high. Also, people's consumption concept is relatively advanced. The west is situated in the remote area. The defects of the geographical environment make its economic development lag behind. The population is rare, while the consumption market development is unsound. Moreover, people's consumption concept is relatively lagging. On this basis, this paper came up with the hypothesis 3: There is heterogeneity of regions between aging population and the upgrade of the consumption structure. Moreover, the upgrade of the consumption structure in the east has the strongest positive effect.

## Empirical model and data description

### The description of the bilateral stochastic cutting-edge model

Through the above-mentioned analysis, it is concluded that there is the mutually exclusive effect of positive and negative directions in the upgrade of the consumption structure caused by aging population. Hence, based on the research idea of Kumbhakar and Parmeter (27), this paper constructed the bilateral stochastic cutting-edge model:


(1) $$\begin{array}{l} Upgrade_{it}=i(x_{it})+w_{it}-u_{it}+\varepsilon_{it}=i(x_{it})+\xi_{it}\\ \;=x_{it}\delta +\xi_{it} \end{array}$$

in which *Upgrade*_*it*_ is the consumption structure level; *x*_*it*_ refers to a series of control variables affecting the upgrade of the consumption structure, including per capita disposable incomes, deposit balance, consumption tendency, child-rearing ratio, urbanization level, industrial development level, social safeguard level, Internet penetration rate, and telephone popularity rate. δ is the parameter vector to be estimated; *i*(*x*_*it*_) refers to the cutting-edge industrial structure level; ξ_*it*_ denotes the compound residual term, ξ_*it*_ = *w*_*it*_−*u*_*it*_+ε_*it*_, in which, ε_*it*_ is the stochastic error term, showing the unobservable factors on the consumption structure level. Since compound residual term ξ_*it*_ is possible to be equal to 0, it will result in the bias in OLS estimation results. As *w*_*it*_ ≥ 0, it means the aging population can promote the upgrade of the consumption structure; as *u*_*it*_ ≥ 0, it means that aging population is not good for the upgrade of the consumption structure; as *w*_*it*_=0, *u*_*it*_ ≥ 0 or *u*_*it*_= 0, *w*_*it*_ ≥ 0, the model means the bilateral stochastic cutting-edge model. As *w*_*it*_ = *u*_*it*_= 0, the model is the OLS model.

Through the Formula (1), the actual effect of aging population for the upgrade of the consumption structure is the result under the combined action of positive and negative bilateral effect of aging population: Aging population promotes the upgrade of the consumption structure so that the consumption structure level is higher than the cutting-edge consumption structure level, while aging population obstructs the upgrade of the consumption structure so that the consumption structure level is lower than the cutting-edge consumption structure level. The net effect based on the combined influence of promotion and obstruction can measure the deviation degree of the practical consumption structure level.

Due to the bias in OLS estimation, to estimate parameter δ and residual terms *w*_*it*_ and *u*_*it*_, this paper used the maximum likelihood estimation (MLE) to get the effective estimation results. To this end, ξ_*it*_ is the compound residual term and its distribution should satisfy the following conditions: the stochastic error term is mutually independent; ε_*it*_ observes normal distribution. In other words, ε_*it*_~ iidN(0,$\sigma_{\varepsilon }^{2}$), *w*_*it*_ and *u*_*it*_ observe the exponential distribution, namely *w*_*it*_~ iidEXP($\sigma_{w}\sigma_{w}^{2}$),*u*_*it*_~ iidEXP($\sigma_{u}\sigma_{u}^{2}$). The error term and upgrade characteristic of the consumption structure *x*_*it*_ are irrelevant. Based on the distribution assumption of the above-mentioned residual term, the probability density function of the compound residual term § it is deduced below:


(2) $$\begin{array}{l} f(\xi_{it})=\frac{exp(\alpha_{it})}{\sigma_{w}+\sigma_{u}}\Phi (\gamma_{it})+\frac{exp(\alpha_{it})}{\sigma_{w}+\sigma_{u}}\Phi (\gamma_{it})\int_{-\xi_{it}}^{\infty }\varphi (x)\;\\ \;=\frac{exp(\alpha_{it})}{\sigma_{w}+\sigma_{u}}\Phi (\gamma_{it})+\frac{exp(\alpha_{it})}{\sigma_{w}+\sigma_{u}}\varphi (\zeta_{it}) \end{array}$$

In Formula (2), Φ(∙) is the accumulative distribution function of the standard normal distribution. φ(∙) is the probability density function and other parameters are set up as $\alpha_{it}=\frac{\alpha_{v}^{2}}{{2\sigma }_{w}^{2}}+\frac{\xi_{i}}{\sigma_{w}};\beta_{it}=\frac{\alpha_{v}^{2}}{{2\sigma }_{u}^{2}}-\frac{\xi_{i}}{\sigma_{u}};\gamma_{it}=\frac{\xi_{i}}{\sigma_{v}}-\frac{\sigma_{v}}{\sigma_{u}}$;$\delta_{it}=\frac{\xi_{i}}{\sigma_{v}}-\frac{\sigma_{v}}{\sigma_{w}}$. Furthermore, based on the estimation of the above-mentioned parameters, the MLE in n observational value samples can be written as follows:


(3) $$\begin{array}{l} \ln\;L\left(X,\pi \right)=-n\ln\left(\sigma_{w}+\sigma_{v}\right)+\overset{n}{\underset{i=1}{\sum }}\ln\left[e^{it}\Phi \left(\gamma_{it}\right)+e^{it}\Phi \left(\zeta_{it}\right)\right] \end{array}$$

in which π= [β,σ_*v*_, σ_*w*_,σ_*u*_], the maximum likelihood function (3) can be used to get all parameter values of MLE to further deduce the conditional density function of *w*_*it*_ and *u*_*it*_:


(4) $$\begin{array}{l} f\left(w_{it}|\xi_{it}\right)=\frac{\left(\frac{1}{\sigma_{u}}+\frac{1}{\sigma_{w}}\right)exp\left[-\left(\frac{1}{\sigma_{u}}+\frac{1}{\sigma_{w}}\right)w_{it}\right]\Phi \left(\frac{w_{it}}{\sigma_{v}}+\zeta_{it}\right)}{exp\left(\beta_{it}-\alpha_{it}\right)\left[\Phi \left(\zeta_{it}\right)+exp\left(\alpha_{it}-\beta_{it}\right)\Phi \left(\gamma_{it}\right)\right]} \end{array}$$

(5) $$\begin{array}{l} f\left(u_{it}|\xi_{it}\right)=\frac{\left(\frac{1}{\sigma_{u}}+\frac{1}{\sigma_{w}}\right)exp\left[-\left(\frac{1}{\sigma_{u}}+\frac{1}{\sigma_{w}}\right)u_{it}\right]\Phi \left(\frac{u_{it}}{\sigma_{v}}+\zeta_{it}\right)}{\Phi \left(\gamma_{it}\right)+exp\left(\alpha_{it}-\beta_{it}\right)\Phi \left(\gamma_{it}\right)} \end{array}$$

This paper focused on the positive and negative bilateral effect of aging population for the upgrade of the consumption structure. As a result, based on Formula (4) and Formula (5), the degree that aging population promotes or obstructs the upgrade of the actual consumption structure deviates the upgrade of the cutting-edge consumption structure. What is more, this paper changed the deviation degree's absolute value that aging population affects the consumption structure level into the percentage that is higher or lower than the upgrade level of the cutting-edge consumption structure. The transformed estimation value is estimated as follows:


(6) $$E(1-e^{-w_{it}}|\xi_{it})=1-\frac{\left(\frac{1}{\sigma_{u}}+\frac{1}{\sigma_{w}})[\Phi (\gamma_{it})+exp(\beta_{it}-\alpha_{it})exp(\frac{\sigma_{\upsilon }^{2}}{2}-\sigma \upsilon \zeta_{it})\Phi (\zeta_{it}-\sigma_{\upsilon })\right]}{\left[1-(\frac{1}{\sigma_{u}}+\frac{1}{\sigma_{w}})]exp(\beta_{it}-\alpha_{it})[\Phi (\zeta_{it})+exp(\alpha_{it}-\beta_{it})\Phi (\gamma_{it}\right)}$$

(7) $$E(1-e^{-u_{it}}|\xi_{it})=1-\frac{\left(\frac{1}{\sigma_{u}}+\frac{1}{\sigma_{w}})[\Phi (\zeta_{it})+exp(\beta_{it}-\alpha_{it})exp(\frac{\sigma_{\upsilon }^{2}}{2}-\sigma \upsilon \zeta_{it})\Phi (\gamma_{it}-\sigma_{\upsilon })\right]}{\left[1-(\frac{1}{\sigma_{u}}+\frac{1}{\sigma_{w}})][\Phi (\zeta_{it})+exp(\alpha_{it}-\beta_{it})\Phi (\gamma_{it}\right)}$$

Furthermore, the net effect (NE) of aging population for the upgrade of the consumption structure can be deduced from Formula (6) and Formula (7):


(8) $$\begin{array}{l} NE=E(1-e^{-w_{it}}|\xi_{it})-E(1-e^{-u_{it}}|\xi_{it})\\ \;=E(e^{-u_{it}}-e^{-w_{it}}|\xi_{it}) \end{array}$$

### Variable selection and data source

Based on the above-mentioned measurement model and data availability, relevant variables can be set up as below:

#### Explained variable: The upgrade level of the consumption structure

It is believed that when residents' consumption demands are changed from the survival-oriented consumption of food, clothing, and housing to five developmental and enjoyable consumption transformation of traffic communication expenditure, leisure entertainment expenditure, educational expenditure, medical expenditure, and daily service expenditure, it is deemed as the residents' upgrade of consumption structure. Hence, by following and measuring residents' consumption level index—residents' average consumption tendency (the ratio between residents' average consumption expenditure and disposable incomes in each region every year), this paper defined the ratio of traffic communication expenditure, leisure entertainment expenditure, educational expenditure, medical expenditure, and daily service expenditure in residents' disposable income (service product consumption tendency) as the index to measure residents' upgrade of the consumption structure.

#### Explaining variable: Aging population level

This paper applied the ratio of aging population over 65 years old in the total population. Meanwhile, to further verify the empirical results in this paper, the old-age dependency ratio variable could be used for the robustness test.

By learning from existing relevant studies, control variables selected in this paper mainly included (1) child-rearing ratio which can be measured by the ratio between child population below 14 years old and labor population; (2) urbanization level which can be measured by the ratio of urban population in total population; (3) residents' per capita disposable incomes; (4) residents' deposit balance; (5) social safeguard level; (6) industrial developmental level which can be measured by the ratio between the level of the tertiary industry and gross domestic product (GDP); (7) Internet penetration rate.

### Data source and descriptive analysis

This paper selected panel data of 31 provinces in China from 2001 to 2018 as the research samples. Relevant data mainly came from annual data of each province from the National Bureau of Statistics, the Chinese Statistical Yearbook, the Chinese Residents' Survey Yearbook, the Yearbook of Chinese Population and Employment Statistics, and the Statistical Report of Chinese Internet Development Status in previous years. Some deficient data could be supplemented by calculating the annual average growth rate and combining with other relevant data. The main variable information is stated in Table 1.

**Table 1:** Descriptive statistics of main variables (*N* = 558).

	**Variables**	**Samples**	**Mean**	**Standard deviation**	**Min**.	**Max**.
Dependent variables	Upgrade of consumption structure	558	0.263	0.209	0.014	1.427
Independent variables	Elderly population ratio	558	0.094	0.031	0.007	0.53
	Old-age dependency ratio	558	0.126	0.029	0.067	0.227
Control variables	Per capita disposable incomes	558	13,000	5844.743	5267.42	43,000
	Year-end balance	558	8188.708	8409.492	62.657	60,000
	Child population ratio	558	0.181	0.057	0.05	0.691
	Urbanization level	558	0.48	0.171	0.143	0.896
	Ratio of the tertiary industry	558	0.452	0.088	0.298	0.831
	Internet penetration rate	558	0.308	0.221	0.015	0.87
	Social safeguard level	558	0.027	0.021	0.001	0.178

*Data source: Chinese Statistical Yearbook, Chinese Residents' Survey Yearbook, Yearbook of Chinese Population and Employment Statistics, and Statistical Report of Chinese Internet Development Status in previous years*.

## Empirical results and analyses

### The estimation of the bilateral stochastic cutting-edge model

#### The reference result

Based on the above-mentioned information, this paper estimated the bilateral effect of aging population for the upgrade of the consumption structure from the perspective of Formula (1). The estimation results can be shown in Table 2. Among them, model 1 in the second row refers to the simple OLS estimation. The values from third row to fifth row refer to MLE estimation results, in which the third row is the uncontrollable time and regional fixed effect. The fourth row is the controllable regional fixed effect, and the fifth row is the controllable time fixed effect. The sixth row is the simultaneous time and regional fixed effect. On this basis, the elderly population ratio over 65 years old was introduced while considering the effect of aging population for the upgrade of the consumption structure, in which the seventh row might only consider the unilateral estimation result of aging population for the negative effect of upgrading the consumption structure. The eighth row might be the unilateral estimation result of aging population for the positive effect of upgrading the consumption structure. The ninth row might be the estimation result that might simultaneously consider the bilateral effect of aging population for upgrading the consumption structure. By comparing the maximum likelihood ratio of each model in Table 2, it could be found that the estimation result of model 7 was the most robust (maximum likelihood ratio); thus, the subsequent variance decomposition and effect estimation should be based on model 7.

**Table 2:** Basic estimation results of bilateral stochastic cutting-edge model (*N* = 558).

**Variables**	**OLS**	**MLE (Uncontrollable time and regional effect)**	**MLE (Controllable regional effect)**	**MLE (Controllable time effect)**	**MLE (Controllable time and regional effect)**	**Negative effect of aging population**	**Positive effect of aging population**	**Bilateral effect of aging population**
	**Model 1**	**Model 2**	**Model 3**	**Model 4**	**Model 5**	**Model 6**	**Model 7**	**Model 8**
Child population ratio	−0.075* (−1.85)	0.0655* (1.89)	0.0704** (1.97)	−0.0801** (−2.06)	−0.0813** (−2.07)	−0.0749** (−1.97)	−0.0956** (−2.15)	−0.0956** (−2.11)
Per capita disposable incomes	−1.0092*** (−53.35)	−0.9991*** (−61.35)	−0.9994*** (−62.25)	−0.9975*** (−72.61)	−0.9975*** (−74.19)	−0.9976*** (−74.05)	−0.9995*** (−67.19)	−0.9995*** (−67.19)
Year-end balance	0.09*** (7.77)	0.13*** (12.9)	0.12*** (12.24)	0.09*** (9.19)	0.08*** (8.41)	0.08*** (8.35)	0.07*** (7.12)	0.07*** (7.11)
Urbanization level	0.77*** (21.09)	0.87*** (25.58)	0.87*** (25.66)	0.84*** (25.661)	0.83*** (25.65)	0.83*** (26.38)	0.79*** (20.71)	0.79*** (20.68)
The development level of tertiary industry	0.69*** (5.48)	0.66*** (6.21)	0.55*** (4.86)	0.82*** (9.73)	0.71*** (7.59)	0.71*** (7.48)	0.63*** (7.88)	0.63*** (7.85)
Internet penetration rate	0.75*** (6.79)	1.16*** (16.78)	1.19*** (17.17)	0.51*** (5.97)	0.56*** (6.33)	0.57*** (6.47)	0.65*** (7.6)	0.65*** (7.47)
Social safeguard level	−1.57*** (−2.76)	0.48 (1.26)	0.68* (1.71)	−1.94*** (−3.77)	−1.75*** (−3.41)	−1.69*** (−3.44)	−1.61*** (−3.04)	−1.61*** (−2.99)
Regional fixed effect	YES	NO	YES	NO	YES	YES	YES	YES
Time fixed effect	YES	NO	NO	YES	YES	YES	YES	YES
Constant term	7.36*** (30.42)	6.73*** (34.82)	6.85*** (35.01)	7.26*** (40.67)	7.37***(41.1)	7.39*** (41.1)	7.47*** (44.59)	7.47*** (44.44)
Stochastic error term		−2.75*** (−10.35)	−2.82*** (-7.73)	−2.99*** (−11.28)	−3.04*** (−9.2)	−3.01*** (−9.57)	−4.79*** (−3.23)	−4.79*** (−3.23)
**Negative effect**
Aging population							−1.54*** (−4.36)	−1.54*** (−4.33)
Constant term		−2.56*** (−18.1)	−2.55*** (−16.9)	−2.87*** (−17.7)	−2.87*** (−16.2)	−2.88*** (−15.9)	−6.49*** (−7.3)	−6.49*** (−7.2)
**Positive effect**
Aging population						0.19 (1.13)		0.0006 (0.0033)
Constant term		−1.87*** (-25.2)	−1.86*** (−23.5)	−1.84*** (−28.3)	−1.84*** (−28.1)	−1.38*** (−3.4)	−1.81*** (−34.6)	−1.81*** (−4.4)
Likelihood ratio		3.28	5.79	100.82	108.02	109.32	134.46	134.46
*P*-value		0.000	0.016	0.000	0.000	0.000	0.000	0.000
Adjusted R	0.95							
Sample size	558	558	558	558	558	558	558	558

Data in the bracket refer to t-value. ^*^, ^**^, and ^***^, respectively, represent significance in 10, 5, and 1%.

#### Variance decomposition: Measurement of the positive and negative effects

According to the regression result of model 8 in Table 2, the promotion and obstruction effect of aging population for the upgrade of the consumption structure is illustrated in Table 3. The estimation results show that aging population exactly shows the positive and negative bilateral effect for the upgrade of the consumption structure. This is consistent with the theoretical hypothesis in this paper, in which the positive effect's estimation coefficient of aging population for the upgrade of the consumption structure was 0.1632 and the negative effect's estimation coefficient was 0.0727, showing that the positive effect was obviously higher than the negative effect. The comprehensive net effect was 0.0905. The further analysis showed that in the influence ratio, the total variable of the stochastic error term that could not be explained by aging population was 0.032 while the ratio in the effect's total variable of the upgrade of the consumption structure to be explained by the bilateral effect of aging population was up to 99.78%, showing that the total utility of aging population explained most parts of the total variable in the upgrade of the consumption structure and verified that aging population exerted the influence on the upgrade of the consumption structure. In total utility of aging population for the upgrade of the consumption structure, aging population's positive effect ratio for the upgrade of the consumption structure was up to 83.43%. The negative effect ratio accounted for 16.57%. The overall results indicated that aging population's positive effect for the upgrade of the consumption structure was greater than that of the negative effect. In this way, the overall upgrade level of the consumption structure was higher than that of the cutting-edge upgrade level of the consumption structure.

**Table 3:** Variance decomposition: the positive effect and negative effect of aging population (*N* = 558).

	**Variable meaning**	**Symbols**	**Measurement** **coefficient**
Influence of aging population	Stochastic error term	σ_*v*_	0.0083
	Negative effect	σ_*u*_	0.0727
	Positive effect	σ_*w*_	0.1632
Variance decomposition	Total stochastic error term	$\sigma_{v}^{2}+\sigma_{u}^{2}+\sigma_{w}^{2}$	0.032
	The ratio with the combined influence of positive and negative effect in total variance	$\frac{\sigma_{u}^{2}+\sigma_{w}^{2}}{\sigma_{v}^{2}+\sigma_{u}^{2}+\sigma_{w}^{2}}$	0.9978
	Negative effect ratio	$\frac{\sigma_{u}^{2}}{\sigma_{u}^{2}+\sigma_{w}^{2}}$	0.1657
	Positive effect ratio	$\frac{\sigma_{w}^{2}}{\sigma_{u}^{2}+\sigma_{w}^{2}}$	0.8343

The influence degree of aging population for the upgrade of the consumption structure. To further change aging population's deviation degree for the upgrade of the consumption structure level into the percentage that is higher than the cutting-edge upgrade level of the consumption structure level, based on Formulas (6)–(8), it, respectively, represented the positive and negative effects of aging population in this paper. The upgrade of the consumption structure deviated the cutting-edge upgrade level of the consumption structure's net effect percentage distribution characteristics. The results can be shown in Table 4. It can be found from the estimation results of Table 4 that on average, aging population promoted 14.04% for the upgrade of the consumption structure and obstructed 6.18% for the upgrade of the consumption structure. The net effect of their mutual influence made the actual upgrade level of the consumption structure slightly higher than 7.86% of the cutting-edge upgrade level of the consumption structure. In other words, if the cutting-edge upgrade level of the consumption structure is assumed as 100%, the ultimate actual level is 107.86%. Details from the fourth row to the sixth row reported the distribution status of the aging population's positive effect, negative effect, and their net effect. The findings showed the aging population's influence for the upgrade of the consumption structure showed the significant difference. Among them, the estimation results of 25 percentiles indicated that under the combined role of aging population's positive and negative effects, the upgrade of the consumption structure in 1/4 provinces was obstructed so that the actual net effect was lower than 0.75% of the cutting-edge level. The reason is that the economic development of 1/4 provinces lags behind and the industrial development is unsound, showing the small stimulation role for the elderly population consumption. For 50 percentiles, the positive effect of 1/4 provinces exceeded the negative effect so that the ultimate net effect was positive. The actual net effect was higher than 5.78% of the cutting-edge level. For 75 percentiles, the positive effect of 1/4 provinces surpassed the negative effect so that the positive net effect was further improved. The ultimate actual net effect was higher than 14.56% of the cutting-edge level.

**Table 4:** Effect estimation of aging population's positive and negative effect affecting the upgrade of the consumption structure (*N* = 558).

**Variables**	**Mean**	**Variation**	**25 percentiles**	**50 percentiles**	**75 percentiles**
Positive effect	14.04	11.53	5.09	9.94	18.98
Negative effect	6.18	4.53	3.71	4.7	6.03
Net effect	7.86	13.65	−0.75	5.78	14.56

**Figure 1:**
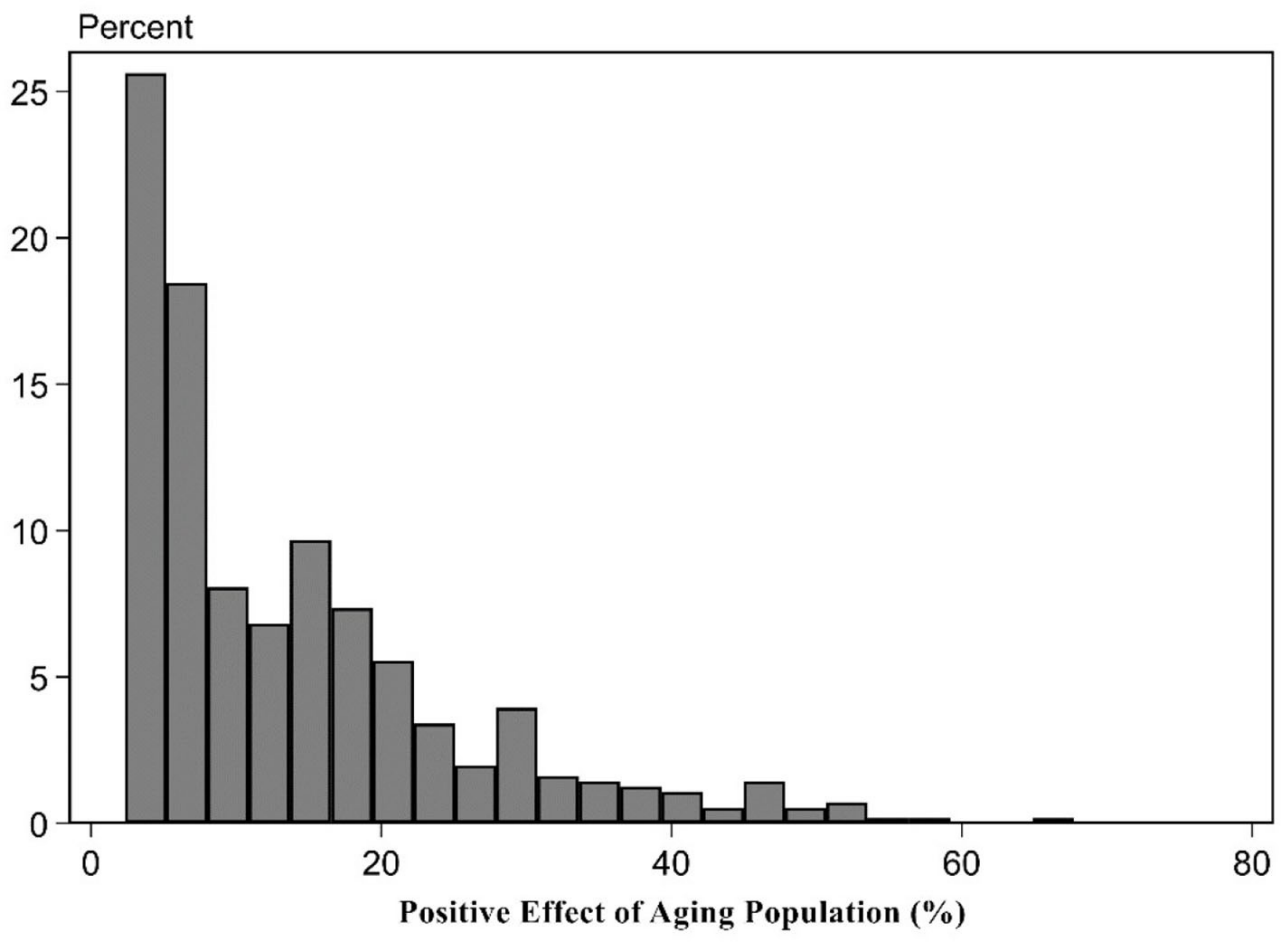
Positive effect of aging population.

**Figure 2:**
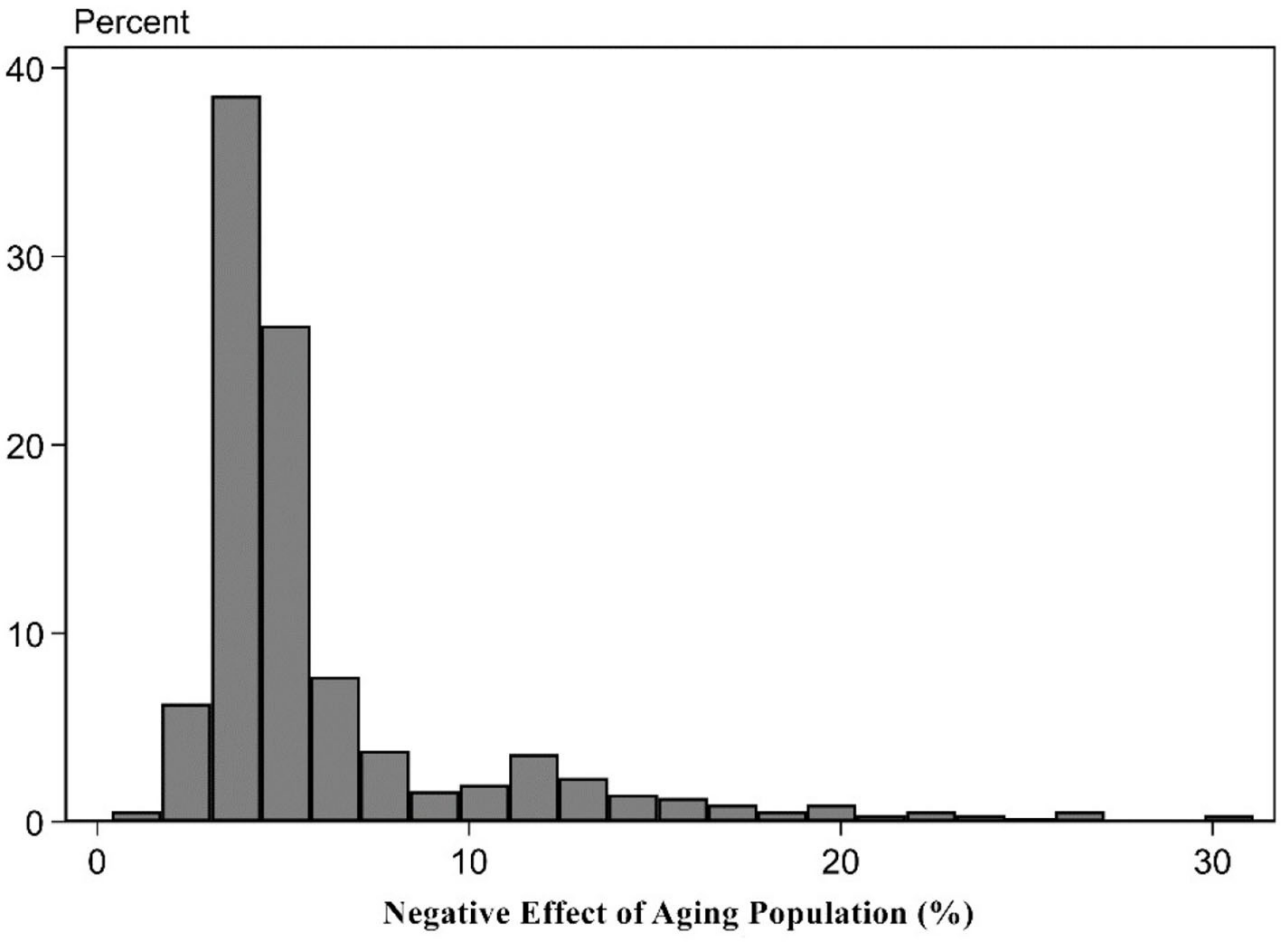
Negative effect of aging population.

**Figure 3:**
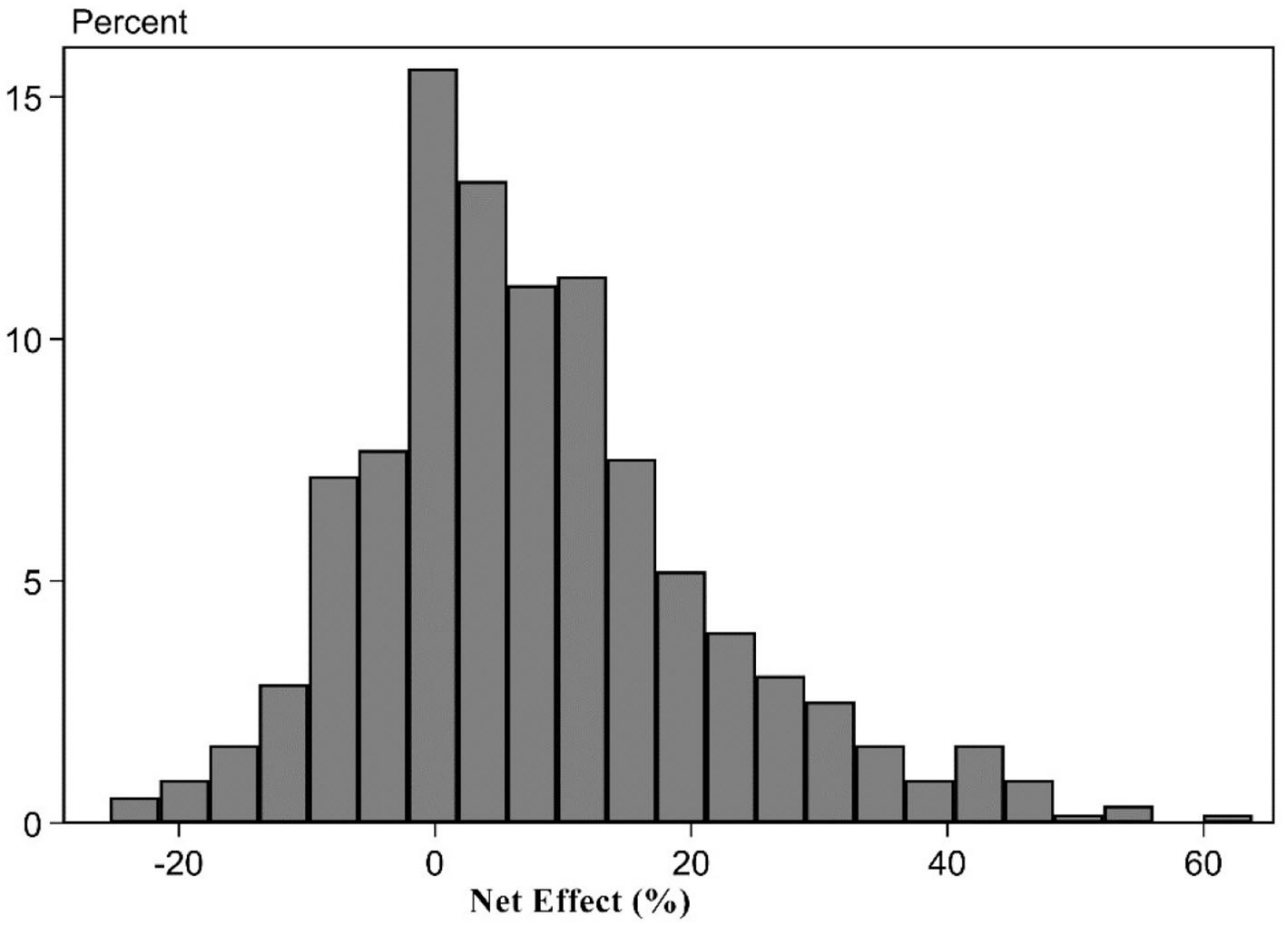
Net effect of aging population.

This paper displayed the chart of frequency distribution among three of them to intuitively display the positive, negative, and net effect distribution situations for aging population affecting the upgrade of the consumption structure (Figures 1–3). Figures 1, 2 indicated the positive effect and negative effect of aging population performing the distribution characteristics of rightward trailing. Among them, Figure 2 stated that aging population's negative obstruction effect should disappear around 30%. Figure 1 showed that the positive promotion effect of aging population still showed the trailing phenomenon around 50%, showing that the promotion role of aging population for the upgrade of the consumption structure was slightly large. Figure 3 indicated that according to the distribution comparison of positive effect and negative effect of aging population, aging population's negative effect was obviously larger than the ratio of the positive effect.

### The time characteristic analysis of aging population net effect

Table 5 exhibits the time distribution characteristics of aging population for the upgrade of the consumption structure. As shown in Table 5, after entering the 21st century, China has entered aging population society. The estimation results of 2001–2008 displayed that the net effect of aging population always showed the positive value, showing the gradually rising trend. However, in the stage of the global financial crisis in 2008, the net effect of aging population dropped. Moreover, since 2008, the net effect of aging population was rising with fluctuations. However, affected by the economic crisis trauma, the growth speed of the overall net effect had the slow speed. However, the overall net effect in previous years always remained the positive value, showing that aging population's promotion effect for the upgrade of the consumption structure always occupied the advantageous position. Meanwhile, according to the quantile results, when each year remained the low quantile level, aging population's upgrade of the consumption structure had the larger obstruction role. Below 25 percentiles, aging population's net effect in half of years should be the negative value. However, with the constant increase in aging population level, the promotion role of aging population for the upgrade of the consumption structure should be more advantageous. Below 75 percentiles, aging population's net effect should be the positive value, showing the aging population level kept enhancing and it would be good for upgrading the consumption structure as a whole.

**Table 5:** Annual estimation of aging population for the net effect of the upgrade of the consumption structure (*N* = 558).

**Years**	**Mean**	**Variation**	**25 percentiles**	**50 percentiles**	**75 percentiles**
2001	1.49	16.43	−10.38	0.1	11.05
2002	5.28	15.72	−1.18	5.21	16.95
2003	9.44	13.68	2.66	8.82	17.71
2004	21.67	15.94	12.68	21.23	32.32
2005	6.84	12.71	−2.54	5.23	13.62
2006	7.48	12.98	−2.5	3.1	16.58
2007	8.1	10.24	0.16	7	14.2
2008	0.81	9.32	−6.82	0.55	8.44
2009	3.75	8.73	−1.27	2.23	9.02
2010	3.24	9.08	−2.8	3.12	8.81
2011	4.14	8.2	−0.85	2.9	9.37
2012	5.33	8.64	−0.54	5.79	11.44
2013	33.75	10.11	28.53	33.25	42.66
2014	6.06	10.03	−1.93	4.49	11.96
2015	6.83	9.57	1.11	3.16	12.25
2016	7.9	9.92	0.6	6.66	14.92
2017	5.51	9.63	−0.74	4.28	12.13
2018	3.88	10.5	−3.96	3.62	11.61

### The regional characteristic analysis of aging population's net effect

Table 6 exhibits the regional distribution characteristic of aging population for the upgrade of the consumption structure. As shown in Table 6, it displayed the aging population's net effect results in the east, the middle, and the west of China by regarding each province and geographical orientation as the foundation for division. According to the effect result comparison of three areas, it could be seen that aging population's net effect in the eastern areas reached the maximum, 9.93%, followed by 9.07% in the western areas, and 3.16% in the middle areas. On this basis, the specific aging population's net effect results in 31 provinces could be further gained. Among all provinces, Zhejiang Province had the maximum aging population's net effect which was 32.22%. Twenty-six provinces had the positive aging population's net effect, with the proportion over 4/5. Five provinces had the negative aging population's net effect, with the proportion <1/5. As a whole, aging population's net effect level performed by five provinces including Xinjiang, Jiangxi, Hebei, Hainan, and Heilongjiang remained the lower level. In other words, aging population's net effect level remained the negative or low level in the northeast and southwest.

**Table 6:** Regional estimation of aging population for the upgrade of the consumption structure's net effect (*N* = 558).

**Provinces**	**Mean**
Shanghai	15.77
Beijing	7.37
Tianjin	7.14
Shandong	8.96
Guangdong	12.45
Jiangsu	13.38
Hebei	0.9
Zhejiang	32.22
Fujian	11.06
Liaoning	−0.51
East	9.93
Jilin	−0.5
Anhui	1.36
Shanxi	−4.51
Jiangxi	1.79
Henan	7.08
Hubei	−0.03
Hunan	18.37
Heilongjiang	1.01
Middle	3.07
Yunnan	7.67
Inner Mongolia	11.61
Sichuan	5.2
Ningxia	16.38
Guangxi	8.54
Xinjiang	2.61
Hainan	0.48
Gansu	11.27
Tibet	−1.09
Guizhou	6.57
Chongqing	11.93
Shaanxi	12.86
Qinghai	16.33
West	9.16

By combining with 7th National Population Census Data Results, the elderly population ratio over 65 years old was 13.5%. Among provinces with the low aging population's net effect level, the elderly population over 65 years old in Liaoning (17.42%), Jilin (15.61%), Heilongjiang (15.61%), Hubei (14.59%), and Hebei (13.92%) was higher than the national level. The value of Shanxi (12.9%), Jiangxi (11.89%), Tibet (5.67%), and Hainan (10.43%) was lower than the national level. On the whole, except for Tibet, aging population degree of other provinces remained the relatively high level but did not gain the silver hair economic dividend brought by aging population. The reason is that the northeast including Shanxi is always the heavy industrial base, energy base, and granary of China. In the period before the foundation of China, it has contributed a huge power for China's development and it was once the most developed area in China, showing that northeast has had some economic foundation. However, the corresponding heavy industry occupies an important position in northeast. All the times, the northeast does not make a breakthrough progress in exploring economic transformation and development of the service industry is not perfect, so it cannot effectively develop the elderly market. This may restrict the consumption structure development of elderly population. What is more, the northeast and southwest have the severe brain drain phenomenon. Particularly, in the southwest like Jiangxi, Tibet, and Hainan, lots of young talents stay outside, resulting in intensifying the difficulty of economic development in both areas. There are many adverse conditions in the geographical position and economic development environment. All of these are not good for the economic development consumption growth. Hubei's industrial strengths are mainly concentrated on the high-tech industry. Hebei's advantageous industry mainly includes agriculture. Relatively speaking, it has the benign foundation to develop the service industry, but the development of relevant industries is still unsound.

### Bilateral effect estimation with different aging degrees

Through the above-mentioned empirical analysis, it could be observed that the aging population's positive promotion role for the upgrade of the consumption structure was greater than that of the obstruction role. On this basis, this paper further classified the aging population degrees and explored the constant deepening influence of the aging population degrees on the upgrade of the consumption structure. According to the international standard, when the elderly proportion ratio over 65 years old exceeds 7%, it means that the country enters the aging society. When this ratio surpasses 14%, it implies that the state enters the deep aging society. When this ratio exceeds 21%, it means that it enters the ultra-aging society. Since the maximum elderly population over 65 years old did not exceed 21%, this paper divided aging degrees into 0–7, 7–14%, and 14–21%. It could be observed from the empirical results in Table 7 that as remaining the 0–7% interval of aging population degrees, aging population could positively promote the upgrade of the consumption structure (1%). With the deepening of aging population, when the aging degrees reached 14–20%, aging population positively facilitated the upgrade of the consumption structure (9.05%), showing that the constant deepening of the aging degrees, the aging population's promotion role on the upgrade of the consumption structure kept enhancing.

**Table 7:** Bilateral effect estimation of different aging degrees (*N* = 558).

**Elderly population ratio over 65 years old**	**Mean**	**Variance**	**25 percentiles**	**50 percentiles**	**75 percentiles**
0–7%	1	14.03	−9.62	1.03	10.26
7–14%	8.72	13.35	−0.49	6.69	15.36
14–21%	9.05	10	2.34	4.77	15.35
Total	7.77	13.54	−0.78	5.77	14.51

### Bilateral effect degree of different urbanization degrees

Generally speaking, with the enhancement on the urbanization level in an area, on the one hand, it is good for young people to gather to drive consumption growth in this area, improve the regional consumption structure, and reversely neutralize the negative effect brought by aging population. On the other hand, the enhancement on the urbanization level means the comprehensive enhancement of the regional economic developmental level, the developmental level of tertiary industry, and elderly product development degree. The enhancement on the urbanization level can provide more convenient, safer, and more abundant consumption experience for older adults. This can positively stimulate consumption growth of older adults and upgrade the consumption structure for the elderly. To verify this guess, this paper divided the urbanization degree into three levels and verified aging population's effect for the upgrade of the consumption structure under different urbanization degrees.

As illustrated in Table 8, under the circumstance with the low urbanization level, aging population's promotion role for the upgrade of the consumption structure was only 1%. With the enhancement of the urbanization level, when the regional urbanization level reached 30–70%, aging population promoted the upgrade of the consumption structure (8.72%); when regional urbanization level surpassed 70%, aging population's promotion role for the upgrade of the consumption structure was 9.05%, showing that with the constant enhancement on the urbanization level, aging population's promotion role for the upgrade of the consumption structure was enhanced accordingly. Improving the urbanization rate could effectively improve aging population's promotion role for consumption and reduce the inhibition role for aging population.

**Table 8:** Bilateral effect estimation of different urbanization levels (*N* = 558).

**Urbanization level**	**Mean**	**Variance**	**25 percentiles**	**50 percentiles**	**75 percentiles**
0–30%	1	14.03	−9.62	1.03	10.26
30–70%	8.72	13.35	−0.49	6.69	15.36
70%–1	9.05	10	2.34	4.77	15.35
Total	7.77	13.54	−0.78	5.77	14.51

## Robustness test

Old-age dependency ratio refers to the specific value between the elderly in non-labor age population and labor age population or it is called the elderly burden coefficient, showing the number of the elderly to be burdened by labor age population in society. This can reveal the social aging degree to some extent. To verify robustness of estimation results, on the basis of the original estimated results, the elderly population ratio above 65 years old was replaced as old-age dependency ratio. Then, the bilateral effect of aging population for the upgrade of the consumption structure was estimated again.

The paper exhibited the estimation result for upgrading the consumption structure by regarding old-age dependency ratio as the main explaining variable. To save the length, the paper directly displayed the bilateral effect result after variance decomposition, as shown in Table 9. The estimation results indicated that old-age dependency ratio showed the positive and negative bilateral effect for the upgrade of the consumption structure, in which old-age dependency ratio showed 0.158 positive effect estimate coefficient for the upgrade of the consumption structure while the negative estimate coefficient was 0.0473. Such a result remained the same with the reference result, verifying the above-mentioned results. Regarding the net effect of old-age dependency ratio, the negative effect of old-age dependency ratio was smaller than that of the positive effect. Similarly, it showed that aging population should be good for 91.68% of the upgrade of the consumption structure. The positive effect of old-age dependency ratio accounted for 91.76%, while the negative effect accounted for 8.24%, showing that old-age dependency ratio's positive role played a dominant role, making the upgrade of the consumption structure positively deviate from the cutting-edge level.

**Table 9:** Variance decomposition: the positive effect and negative effect of old-age dependency ratio.

	**Variable meaning**	**Symbols**	**Measurement coefficient**
Influence of aging population	Stochastic error term	σ_*v*_	0.0497
	Negative effect	σ_*u*_	0.0473
	Positive effect	σ_*w*_	0.158
Variance decomposition	Total stochastic error term	$\sigma_{v}^{2}+\sigma_{u}^{2}+\sigma_{w}^{2}$	0.0297
	Ratio of combined influence of positive effect and negative effect in total variance	$\frac{\sigma_{u}^{2}+\sigma_{w}^{2}}{\sigma_{v}^{2}+\sigma_{u}^{2}+\sigma_{w}^{2}}$	0.9168
	Negative effect ratio	$\frac{\sigma_{u}^{2}}{\sigma_{u}^{2}+\sigma_{w}^{2}}$	0.0824
	Positive effect ratio	$\frac{\sigma_{w}^{2}}{\sigma_{u}^{2}+\sigma_{w}^{2}}$	0.9176

Table 10 shows the positive effect, negative effect, and net effect of old-age dependency ratio for the upgrade of the consumption structure. The findings indicated that with the continuous enhancement on old-age dependency ratio, the positive effect of old-age dependency ratio promoted the upgrade of the consumption structure by 13.61%. The negative effect of old-age dependency ratio reduced the upgrade of the consumption structure by 4.42%. The comprehensive net benefits promoted the actual upgrade of the consumption structure to be higher than that of cutting-edge level by 9.19%.

**Table 10:** Positive effect, negative effect, and net effect estimation of old-age dependency ratio for the upgrade of the consumption structure (*N* = 558).

**Variables**	**Mean**	**Variance**	**25 percentiles**	**50 percentiles**	**75 percentiles**
Positive effect	13.61	11.15	5.48	9.38	17.84
Negative effect	4.42	3.88	2.13	3.39	5.18
Net effect	9.19	12.53	1.56	6.35	14.27

## Conclusion and advice

On the grounds of the above-mentioned analysis, this paper drew the following conclusions: (1) Aging population showed a certain negative and negative bilateral effect for the upgrade of the consumption structure. Moreover, the positive effect of aging population was greater than that of the negative effect. (2) From the perspective of time effect, under the combined role of aging population's positive and negative effects, the upgrade of the consumption structure was promoted by 7.861% on average every year. (3) Aging population's effect for the upgrade of the consumption structure showed regional heterogeneity. The aging population's positive promotion role in the eastern areas reached the maximum, followed by the western areas, and middle areas were the minimum. The reason is that aging population's net effect of middle areas and western areas includes three provinces of northeast and southwestern areas. (4) With the continuous enhancement of aging population and urbanization level, aging population's promotion effect for the upgrade of the consumption structure enhanced with it. For this reason, the paper put forward the following advice:

First, it is necessary to positively develop economy and promote improvement of the consumption structure according to the circumstances. By estimating net effect of aging population in different provinces, it could be found that provinces with the relatively significant aging population's positive effect mainly included areas with the higher economic developmental level including Zhejiang, Shanghai, Guangdong, and Jiangsu. Hence, it is necessary to facilitate consumption growth under the circumstance of keeping deepening aging population, construct domestic circulation, and promote consumption growth. Under the circumstance, it is essential to develop national economy. Only by remaining faster and better economic development, it can effectively facilitate consumption upgrade. The dramatic drop of aging population's negative effect brought by the economic crisis in 2008 also verified the importance of economic development. What is more, economic development and industrial upgrade of each area should be promoted to the point which is reinforced according to circumstances. On the basis of estimating aging population's net effect of each province, aging population's net effect in southwest and northeast should be dominated by the lower net effect or negative effect. For the southwest with the relatively lagging economy, the regional characteristic economy should be developed. Meanwhile, northeast and Shanxi that have been equipped with favorable industrial foundation should drive development of tertiary industry and realize economic transformation. Hubei can depend on the high-tech industrial strength to realize the organic combination of the pension industry and high-tech industry and help to develop silver hair economy.

What is more, it is essential to develop the perfect elderly consumption market and explore the consumption potential of old groups. China has the large population base and fast aging speed (28). The “silver hair economy” inevitably will become the key of future service industry development. Also, the empirical results also indicated that with the continuous enhancement on aging degree, aging population's promotion role for the upgrade of the consumption structure kept enhancing, showing that aging population contains the huge consumption growth potential. Hence, developing elderly caring products and services, spiritual caring, tourist industry, and leisure products for older adults could be developed with pertinence. Meantime, based on medical healthcare, it is essential to develop products with the cross integration of medical pension combination, medical food combination, medical use combination, and medical accommodation combination, so as to promote growth of other consumption expenditure with medical healthcare.

Lastly, it is necessary to accelerate the urbanization development progress and depend on the Internet to comprehensively stimulate consumption growth. The estimation results of Table 2 found that the urbanization level, Internet penetration rate, and developmental level of tertiary industry showed the significant positive promotion role for the upgrade of the consumption structure. The empirical results also indicated that with the continuous enhancement on the urbanization level, aging population's promotion role for the upgrade of the consumption structure was enhanced. As a result, in future development process, the elderly consumption could be stimulated by keep improving the urbanization level and developmental level of tertiary industry. Meanwhile, the Internet utilization can make consumption realize rapid development through digitalization and networking (29, 30), so as to make safe, convenient, and reliable elderly services. Depending on the Internet technology, safer and more sustainable consumption channels could be created to develop characteristic features of online purchase and door-to-door old consumption and to stimulate elderly consumption growth depending on the sound consumption environment.

## Data availability statement

The original contributions presented in the study are included in the article/supplementary material, further inquiries can be directed to the corresponding author/s.

## Author contributions

YW contributed to conception, design of the study, and wrote the first draft of the manuscript. WZ organized the database. WM performed the statistical analysis. All authors contributed to manuscript revision, read, and approved the submitted version.

## Funding

This study was supported by the National Social Science Fund of China: Research on the Construction of Birth-friendly Employment Support System for Professional Women under the Background of Low Fertility Rate (No. 20BRK001), the Ministry of Education Humanities Social Sciences the Youth Fund Project: Research on Women's Career Life Cycle Movement and Public Policy Orientation Under the Universal Two-child Policy (No. 17YJC840040), and the National Natural Science Foundation of China: The Optimization Design of New Olive Shape Three Pillars for the Sustainable Development of Pension System National Natural Science Foundation of China (No. 71731007).

## Conflict of interest

The authors declare that the research was conducted in the absence of any commercial or financial relationships that could be construed as a potential conflict of interest.

## Publisher's note

All claims expressed in this article are solely those of the authors and do not necessarily represent those of their affiliated organizations, or those of the publisher, the editors and the reviewers. Any product that may be evaluated in this article, or claim that may be made by its manufacturer, is not guaranteed or endorsed by the publisher.

## Footnotes

^1^ https://www.guancha.cn/politics/2020_11_03_570186.shtml

^2^It refers to eight categories of food expenditure, clothing expenditure, housing expenditure, traffic communication expenditure, educational cultural entertainment expenditure, medical expenditure, and daily consumption service expenditure used by National Bureau of Statistics.
